# Solitary colonic metastasis from hepatocellular carcinoma: A case report and synthesis of 15 published cases

**DOI:** 10.1097/MD.0000000000050286

**Published:** 2026-08-21

**Authors:** Zhenxing Zhang, Kewei Ji, Guolin Zhang, Kelong Tao, Guangen Xu, Shan Wang

**Affiliations:** a Department of Gastrointestinal Surgery, Shaoxing People’s Hospital (The First Hospital of Shaoxing University), Shaoxing, China; b Department of Special Examination, Shaoxing People’s Hospital (The First Hospital of Shaoxing University), Shaoxing, China.

**Keywords:** case report, colon carcinoma, colon metastasis, extrahepatic metastases, hepatocellular carcinoma, metastasis

## Abstract

**Rationale:**

Hepatocellular carcinoma (HCC) is a malignant tumor with the potential for intrahepatic and extrahepatic metastatic spread. Although extrahepatic metastases commonly involve the lungs, lymph nodes, bones, and adrenal glands, gastrointestinal (GI) metastasis, particularly colonic metastasis, is extremely rare. Due to its low incidence and nonspecific endoscopic manifestations, colonic metastasis from HCC can be easily misdiagnosed as a primary colorectal neoplasm. Herein, we report a rare case of colonic metastasis from HCC detected during long-term postoperative surveillance and highlight the importance of considering atypical metastatic sites in patients with HCC.

**Patient concerns:**

A 76-year-old male patient with a history of HCC was found to have a colonic lesion during routine postoperative abdominal computed tomography (CT) surveillance. Seven years earlier, he had been diagnosed with HCC and underwent partial resection of liver segment IV. The patient had evidence of previous hepatitis B virus (HBV) exposure and was receiving oral entecavir therapy.

**Diagnoses:**

Contrast-enhanced CT revealed an occupying lesion at the hepatic flexure of the colon. Colonoscopy demonstrated an approximately 4.0-cm elevated lesion with surface congestion and erosion. Biopsy showed necrotic tissue with a small number of atypical cells, initially suggesting metastatic HCC. The patient subsequently underwent right hemicolectomy. Histopathological examination of the resected specimen revealed an elevated tumor in the ascending colon, consistent with metastatic HCC. Immunohistochemical analysis demonstrated positive Hepatocyte expression and negative CK20, CDX2, and Villin expression, supporting hepatic origin and excluding primary colorectal adenocarcinoma.

**Interventions:**

The patient underwent right hemicolectomy for the colonic lesion. Postoperative antiviral therapy with entecavir was continued, and regular surveillance was performed.

**Outcomes:**

The patient recovered uneventfully after surgery and was discharged on postoperative day 10. At the 2-year follow-up, he remained alive without evidence of recurrent disease or newly identified metastatic lesions.

**Lessons:**

Colonic metastasis from HCC is exceptionally rare and may mimic primary colorectal malignancy. The diagnosis requires careful integration of clinical history, pathological morphology, and immunohistochemical findings. Hematogenous dissemination may represent a possible mechanism of colonic involvement. Clinicians should consider gastrointestinal metastasis during long-term surveillance of patients with HCC, especially in regions with a high prevalence of HBV infection.

## 1. Introduction

Hepatocellular carcinoma (HCC) is one of the most common malignant tumors worldwide and represents a major cause of cancer-related mortality. According to global cancer statistics, HCC accounts for a substantial proportion of primary liver cancers, with the highest disease burden observed in East and Southeast Asia, where chronic hepatitis B virus (HBV) infection remains a major etiological factor.^[1,2]^ Despite advances in surveillance programs, antiviral therapy, and treatment strategies, recurrence and metastasis remain major challenges in the long-term management of patients with HCC.

HCC can spread through both intrahepatic and extrahepatic routes. Extrahepatic metastasis occurs in approximately 13.5% to 42% of patients with advanced HCC and is associated with a poor prognosis.^[3]^ The most frequently involved extrahepatic metastatic sites include the lungs, lymph nodes, bones, and adrenal glands.^[3,4]^ In contrast, gastrointestinal (GI) metastasis from HCC is uncommon, and colonic involvement is particularly rare. Because of its low incidence and nonspecific clinical manifestations, colonic metastasis from HCC can mimic primary colorectal malignancy, creating challenges in diagnosis and subsequent treatment decisions.^[5]^

Current clinical practice guidelines recommend regular surveillance after potentially curative treatment of HCC, including periodic imaging examinations, to detect tumor recurrence and metastatic disease at an early stage.^[6,7]^ However, surveillance strategies primarily focus on intrahepatic recurrence and common extrahepatic metastatic sites, whereas rare metastatic patterns such as gastrointestinal involvement may be overlooked. Recognition of atypical metastatic locations is therefore clinically important, especially in regions with a high prevalence of HBV infection where a large number of patients require long-term HCC surveillance.

Although several cases of gastrointestinal metastasis from HCC have been reported, the available evidence mainly consists of isolated case reports and small case series.^[5]^ Comprehensive systematic reviews focusing specifically on GI metastatic patterns from HCC remain limited, and the clinical characteristics, diagnostic approaches, and optimal management strategies for colonic metastasis are not well established.

Herein, we report a rare case of colonic metastasis from HCC occurring 7 years after hepatic resection in a patient with previous HBV exposure receiving antiviral therapy. Through this case and a review of the available literature, we aim to emphasize the importance of considering gastrointestinal metastasis during long-term surveillance of patients with HCC, particularly in HBV-endemic regions.

## 2. Case presentation

### 2.1. Patient history

A 76-year-old man with a history of HBV infection was diagnosed with HCC 7 years before the current admission. He underwent partial resection of liver segment IV for HCC. After surgery, the patient received regular postoperative surveillance at our hospital, including periodic abdominal CT examinations for the detection of tumor recurrence or extrahepatic metastasis. The patient was receiving antiviral therapy with entecavir (0.5 mg once daily) for HBV management.

**Figure 1.**
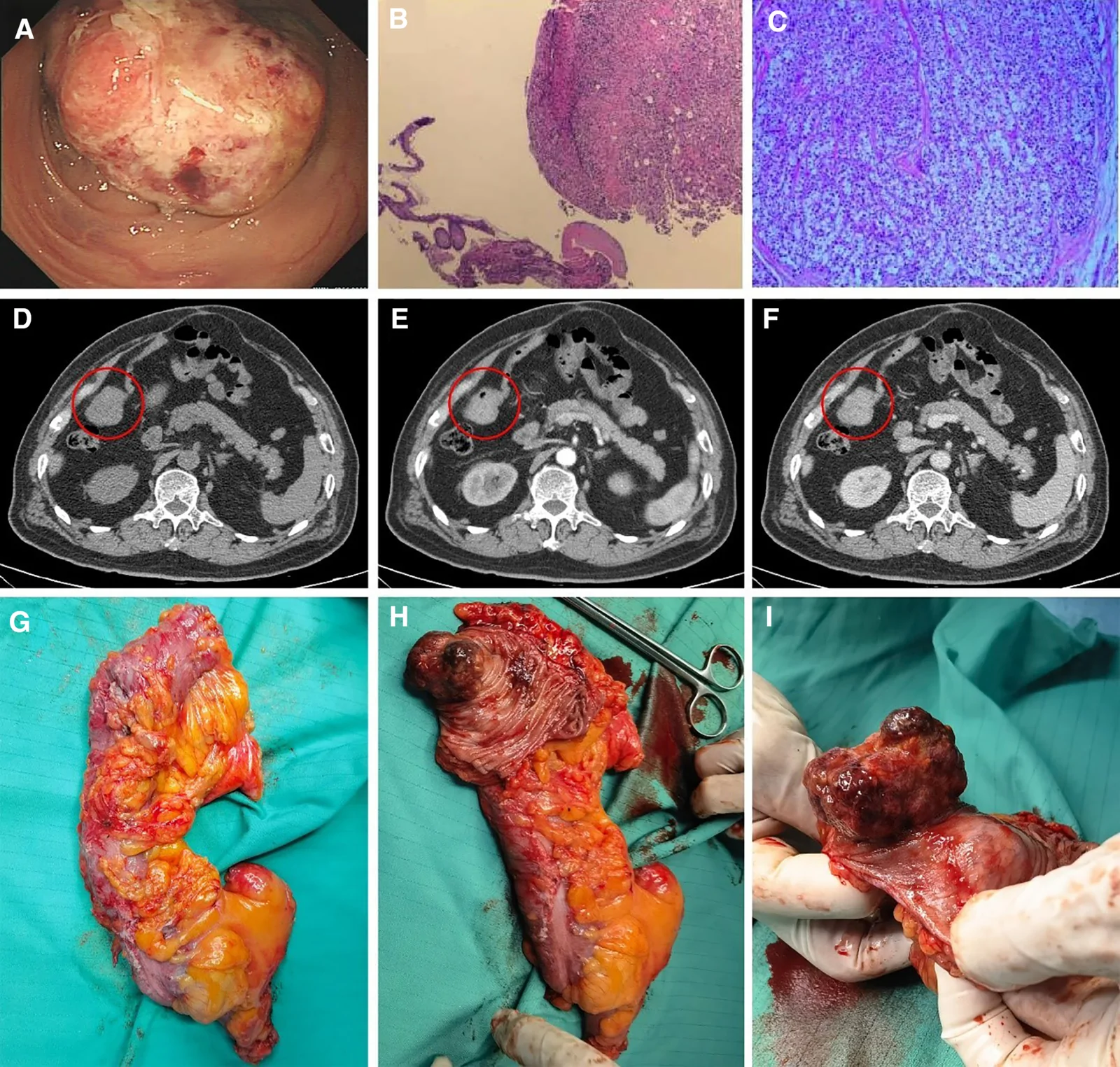
Imaging, endoscopic, pathological, and surgical findings of colonic metastasis from hepatocellular carcinoma. (A) Colonoscopic examination revealed an approximately 4.0-cm elevated lesion with a broad base at the hepatic flexure of the colon. The lesion showed surface congestion and erosion, mimicking a primary colorectal neoplasm and prompting further pathological evaluation. (B) Histopathological and immunohistochemical findings of the colonoscopic biopsy specimen. A small number of atypical cells were identified within necrotic tissue. Positive staining for hepatocyte and negative expression of colorectal epithelial markers supported the diagnosis of metastatic hepatocellular carcinoma rather than primary colorectal cancer. (C) Histopathological and immunohistochemical findings of the surgically resected colonic tumor. The resected specimen demonstrated morphological features consistent with metastatic hepatocellular carcinoma. Hepatocyte positivity confirmed hepatocellular differentiation, while the absence of CDX2 and Villin expression excluded a colorectal origin. (D–F) Preoperative contrast-enhanced computed tomography (CT) demonstrated an occupying lesion at the hepatic flexure of the colon with circumferential bowel wall thickening and enhancement, leading to further endoscopic evaluation. No obvious intrahepatic recurrence was identified after previous partial resection of liver segment IV. (G–I) Intraoperative findings during laparoscopic right hemicolectomy. A protruding tumor was identified at the hepatic flexure of the colon. No additional metastatic lesions were observed in the liver, pelvic cavity, or peritoneum, suggesting an isolated colonic metastatic lesion from HCC. CT = computed tomography, HCC = hepatocellular carcinoma.

During routine follow-up abdominal CT, an occupying lesion was detected at the hepatic flexure of the colon, accompanied by narrowing of the proximal colonic lumen (Fig. 1D–F). The CT scan also demonstrated postoperative changes associated with previous partial resection of liver segment IV, without obvious intrahepatic recurrence.

The patient had no abdominal pain, gastrointestinal bleeding, changes in bowel habits, or other gastrointestinal symptoms. His abdominal physical examination revealed no remarkable abnormalities. His medical history was otherwise unremarkable, and no significant comorbidities were reported.

### 2.2. Clinical findings and diagnostic assessment

Further colonoscopic examination was performed to evaluate the colonic lesion detected on CT. White-light colonoscopy revealed an approximately 4.0-cm elevated lesion with a broad base at the hepatic flexure of the colon. The lesion showed surface congestion and erosion (Fig. 1A).

At this stage, the differential diagnosis included primary colorectal neoplasm, inflammatory lesions, and metastatic lesions from previous HCC. Considering the patient’s history of HCC, metastatic disease was also considered.

Colonoscopic biopsy demonstrated necrotic tissue with a small number of atypical cells. Based on the patient’s history of HCC and the pathological morphology, metastatic HCC was considered the most likely diagnosis. Immunohistochemical (IHC) staining showed positive expression of Hepatocyte and negative expression of CK20, CK7, EMA, CD68, CK19, AFP, and S-100, with a Ki-67 proliferation index of approximately 10% and negative P53 expression (Fig. 1B). The positive expression of Hepatocyte, a marker associated with hepatocellular differentiation, together with the absence of typical intestinal epithelial markers, supported the possibility that the lesion originated from metastatic HCC rather than primary colorectal adenocarcinoma.

Preoperative laboratory examination revealed severe anemia (hemoglobin 51 g/L). Liver function tests showed slightly decreased alanine aminotransferase (ALT 6.7 U/L), aspartate aminotransferase (AST 13.5 U/L), alkaline phosphatase (ALP 41.7 U/L), total protein (54.9 g/L), and albumin (32.8 g/L). Coagulation function, white blood cell count, and bilirubin levels were within normal ranges. Tumor markers, including carcinoembryonic antigen (CEA, 1.16 ng/mL), alpha-fetoprotein (AFP, 2.38 ng/mL), and carbohydrate antigen 19-9 (CA19-9, <2.00 U/mL), were within normal limits.

Because of the severe anemia, the patient received several transfusions of leukocyte-depleted red blood cells before surgery, and hemoglobin levels increased to 92 g/L before surgical intervention.

### 2.3. Therapeutic intervention

Based on the suspected diagnosis of colonic metastasis from HCC, the patient underwent laparoscopic radical right hemicolectomy combined with tension-free repair of an incisional hernia.

During surgery, extensive intra-abdominal adhesions were observed, particularly around the hepatic hilum and hepatic flexure of the colon. These adhesions were considered to be associated with previous liver resection, cholecystectomy, and appendectomy. The abdominal wall was weakened, and an incisional hernia had developed after previous abdominal surgery.

An elevated tumor was identified at the hepatic flexure of the colon, measuring approximately 4.5*4.0*3.0 cm (Fig.1G–I). No obvious metastatic lesions were observed in the liver, pelvic cavity, or peritoneum. A small amount of ascites was present during surgery.

### 2.4. Histopathological findings

Postoperative pathological examination of the right hemicolectomy specimen revealed an elevated tumor measuring 4.5*4.0*3.0 cm in the ascending colon. Histologically, the tumor infiltrated through the entire intestinal wall. Based on the patient’s previous history of HCC, microscopic morphology, and immunohistochemical findings, the lesion was diagnosed as metastatic HCC.

No vascular invasion, lymphatic invasion, nerve invasion, or bile duct invasion was identified. All surgical margins were negative. Examination of 15 regional lymph nodes (including 2 peri-ileal lymph nodes, 5 ileocecal lymph nodes, and 8 pericolonic lymph nodes) showed no evidence of metastatic involvement.

Further IHC analysis demonstrated positive Hepatocyte expression and negative AFP, CK19, CK20, CDX2, Villin, and SALL4 expression. CK7 showed partial positivity. The proliferation index Ki-67 was approximately 20%, while P53 was negative. Mismatch repair protein expression, including MLH1, MSH2, MSH6, and PMS2, was retained (Fig. 1C).

The positive hepatocyte expression indicated hepatocellular differentiation and supported the diagnosis of metastatic HCC. Meanwhile, the absence of colorectal lineage markers, including CDX2 and Villin, argued against primary colorectal adenocarcinoma. Therefore, combining clinical history, morphological features, and IHC findings, the final diagnosis was confirmed as colonic metastasis from HCC.

### 2.5. Follow-up and outcomes

The patient’s postoperative recovery was uneventful. No postoperative complications occurred, and he was discharged on postoperative day 10. After surgery, the patient continued regular follow-up and antiviral therapy with entecavir. At the 2-year follow-up, the patient remained alive without evidence of local recurrence, intrahepatic recurrence, or newly developed metastatic lesions.

## 3. Discussion

HCC is one of the most common malignant tumors worldwide and is characterized by a high risk of recurrence and metastasis even after curative treatment. The metastatic process of HCC involves complex interactions between tumor biological behaviors, vascular invasion, epithelial–mesenchymal transition (EMT), and the tumor microenvironment, which contribute to disease progression and distant dissemination.^[8,9]^ Extrahepatic metastasis is not uncommon in patients with HCC. However, GI involvement remains rare, and solitary colonic metastasis is particularly uncommon. Most reported cases of GI metastasis from HCC are limited to case reports or small case series, and comprehensive analyses remain scarce.^[3]^ According to our literature review, including the present case, only 16 cases of solitary colonic metastasis from HCC had been reported up to 2022 (Tables 1 and 2).^[10-24]^ The reported metastatic sites included the ascending colon (4 cases),^[10,21,23,24]^ transverse colon (4 cases),^[14–16,22]^ sigmoid colon (4 cases),^[11-13,20]^ rectum (2 cases),^[17,19]^ splenic flexure (1 case),^[18]^ and hepatic flexure (present case). These findings highlight the extreme rarity of isolated colonic metastasis from HCC and the limited understanding of its clinical characteristics and underlying mechanisms.

**Table 1:** Summary of previously reported cases of solitary colonic metastasis from hepatocellular carcinoma, including the present case.

Year	Age (yr)	Sex	Underlying liver disease	Colonic metastatic site	Previous treatment	Interval between primary HCC diagnosis and colonic metastasis (mo)	Survival after diagnosis of colonic metastasis (mo)	Reference
2022	76	Male	HBV infection	Hepatic flexure	Hepatic resection + TACE	84	≥24	Present case
2021	80	Male	HCV infection	Ascending colon	Hepatic resection + TACE + RFA	60	30	Miyauchi et al^[10]^
2020	60	Male	HBV infection	Sigmoid colon	Hepatic resection + TACE + RFA + chemotherapy	120	NR	Yu et al^[11]^
2019	60	Male	HBV infection	Sigmoid colon	TACE	NR	NR	Pham et al^[12]^
2019	70	Male	HBV infection	Sigmoid colon	TACE	NR	NR	Tagliabue et al^[13]^
2017	75	Male	Normal liver	Transverse colon	Hepatic resection	7	12	Nakajima et al^[14]^
2017	60	Female	Normal liver	Transverse colon	No previous treatment	0 (simultaneous)	>12	Tanaka et al^[15]^
2016	47	Male	HBV infection	Transverse colon	Hepatic resection + TAE + TACE	60	>12	Zhu et al^[16]^
2016	82	Female	Liver cirrhosis	Rectum	RFA + TACE	4	5	Ikeda et al^[17]^
2014	50	Female	Cryptogenic cirrhosis	Splenic flexure	Liver transplantation + radioembolization	48	NR	Kohli et al^[18]^
2011	57	Female	HCV infection	Rectum	Hepatic resection	18	NR	Huang et al^[19]^
2010	47	Male	HBV infection	Sigmoid colon	TACE	18	>4	Yoo et al^[20]^
2008	69	Male	Unknown	Ascending colon	Hepatic resection	24	24	Nozaki et al^[21]^
2008	79	Male	HCV infection	Transverse colon	TACE	18	6	Hirashita et al^[22]^
2007	67	Male	Autoimmune hepatitis	Ascending colon	Immunosuppressive therapy	NR	>1	Tapuria et al^[23]^
1999	82	Female	HCV infection	Ascending colon	Hepatic resection + chemotherapy	48	NR	Cosenza et al^[24]^

HBV = hepatitis B virus, HCC = hepatocellular carcinoma, HCV = hepatitis C virus, NR = not reported, RFA = radiofrequency ablation, TACE = transarterial chemoembolization, TAE = transarterial embolization.

**Table 2:** Summary statistics of previously reported cases of solitary colonic metastasis from hepatocellular carcinoma.

Variable	Result
Number of previously reported cases	15
Median age at diagnosis of colonic metastasis	67 yr (range: 47–82 yr)
Male patients	10/15 (66.7%)
Female patients	5/15 (33.3%)
HBV infection	5/15 (33.3%)
HCV infection	4/15 (26.7%)
Liver cirrhosis	2/15 (13.3%)
Normal liver/no underlying liver disease	2/15 (13.3%)
Unknown/other liver disease	2/15 (13.3%)

Data were summarized from 15 previously reported cases of solitary colonic metastasis from hepatocellular carcinoma, excluding the present case.

HBV = hepatitis B virus, HCV = hepatitis C virus.

In the present case, a solitary colonic metastatic lesion was detected at the hepatic flexure of the colon 7 years after partial resection of liver segment IV for HCC during routine postoperative surveillance. The patient had a history of HBV infection and was receiving antiviral therapy with entecavir. HBV infection remains one of the major risk factors contributing to the global burden of HCC, particularly in endemic regions of Asia, where long-term surveillance after curative treatment is particularly important.^[25]^ Among previously reported cases, underlying liver diseases included HBV infection (5 cases), hepatitis C virus (HCV) infection (4 cases), liver cirrhosis (2 cases), normal liver background (2 cases), autoimmune hepatitis, and unknown etiology. Unlike the common patterns of extrahepatic recurrence after HCC resection, which frequently involve multiple metastatic sites,^[26]^ our patient showed no evidence of intrahepatic recurrence or other abdominal metastatic disease, representing an unusual pattern of isolated extrahepatic recurrence.

The metastatic pathways of HCC include hematogenous dissemination, lymphatic spread, direct invasion, and peritoneal implantation. Among these mechanisms, hematogenous dissemination is considered a major pathway responsible for distant metastasis. The development of hematogenous metastasis is a multistep process involving tumor cell invasion into blood vessels, survival in the circulation, extravasation, and colonization of distant organs. Recent studies have demonstrated that vascular invasion, portal vein tumor invasion/tumor thrombus formation, abnormal tumor-associated vasculature, EMT, angiogenesis, extracellular matrix remodeling, and interactions between tumor cells and the surrounding microenvironment contribute to metastatic progression of HCC.^[8,9,27]^ These mechanisms may facilitate the dissemination of circulating tumor cells and increase the probability of metastatic colonization at distant organs.

GI tract involvement from HCC is generally considered to occur mainly through direct invasion from adjacent organs, whereas hematogenous dissemination to the GI tract is relatively uncommon.^[28]^ Anatomical proximity is an important factor influencing the pattern of direct invasion. Ou et al^[29]^ reported that the ascending and transverse colon were the most frequently involved sites in direct invasion from HCC, likely because these segments are anatomically closer to the primary hepatic tumor than other parts of the colon. In addition, intraperitoneal implantation has also been proposed as a possible mechanism for GI metastasis from HCC.

However, the metastatic pattern observed in our case was inconsistent with direct invasion or intraperitoneal implantation. Tumors spreading through these pathways usually first involve the serosal surface and subsequently extend toward deeper layers, with mucosal involvement occurring at a later stage. Yoo et al^[20]^ reported a case of sigmoid colon metastasis from HCC caused by direct invasion, in which colonoscopy did not demonstrate an intraluminal tumor but only revealed a bulging contour caused by external compression. In contrast, our patient presented with an obvious intraluminal protruding mass detected by colonoscopy, and intraoperative findings showed tumor invasion involving the full thickness of the intestinal wall without evidence of peritoneal dissemination or adjacent organ invasion. Furthermore, no recurrent lesions were detected in the liver, pelvis, or peritoneal cavity. Therefore, direct invasion and intraperitoneal implantation were considered less likely, and hematogenous dissemination was considered the most plausible metastatic route.

Although the exact metastatic pathway could not be confirmed, several clinical and pathological findings supported hematogenous dissemination. First, the patient had no intrahepatic recurrence or local invasive lesion, making direct extension unlikely. Second, the metastatic lesion presented as an intraluminal mass rather than an externally compressive lesion, which differs from typical direct invasion patterns. Third, pathological examination showed no lymph node involvement or lymphatic invasion. Finally, the isolated nature of the lesion and the long interval after primary HCC treatment suggested that delayed hematogenous dissemination, potentially related to dormant tumor cells, may have contributed to the development of colonic metastasis.

The mechanism of hematogenous spread of HCC to unusual sites remains incompletely understood. Recent studies have suggested that vascular invasion, portal vein tumor thrombus formation, and abnormal tumor-associated vascular structures may facilitate tumor cell entry into the circulation and promote distant metastasis.^[8]^ Portal venous abnormalities may also contribute to atypical metastatic patterns. Tumor thrombus formation within the portal vein may alter portal circulation and potentially facilitate the migration of tumor cells to the gastrointestinal tract. In addition, locoregional therapies such as transarterial embolization (TAE) and transarterial chemoembolization (TACE) may influence intrahepatic hemodynamics and portal venous pressure, theoretically contributing to unusual metastatic routes.^[12]^ In our patient, previous TACE before hepatic resection may have influenced vascular conditions; however, the association between prior TACE and subsequent colonic metastasis remains speculative and requires further investigation.

The prognosis of patients with extrahepatic metastasis from HCC is generally poor. Previous studies have reported limited survival outcomes among patients with extrahepatic metastatic disease, with Natsuizaka et al reporting a mean survival time of approximately 7 months.^[4]^ For patients with advanced HCC and extrahepatic spread, systemic therapies, including molecular targeted agents and immunotherapy, are currently recommended according to contemporary treatment strategies such as the updated Barcelona Clinic Liver Cancer (BCLC) algorithm.^[30]^ However, surgical resection may still provide clinical benefits in carefully selected patients with isolated, technically resectable extrahepatic metastatic lesions, particularly when intrahepatic disease is absent or well controlled.^[31]^ Fujii et al reported that among patients with hepatocellular carcinoma involving the gastrointestinal tract, those who underwent curative surgical resection achieved a survival time of approximately 9.7 months, suggesting that surgical resection may be a feasible option in carefully selected patients with resectable GI metastases.^[32]^

In our case, the patient underwent complete surgical resection of the solitary colonic metastatic lesion. The postoperative course was uneventful, and the patient remained free of recurrence or new metastatic lesions at the 2-year follow-up. Although longer follow-up is required, this case suggests that surgical resection may be considered for selected patients with isolated gastrointestinal metastasis from HCC, especially when complete resection can be achieved and intrahepatic disease remains controlled. Furthermore, this case emphasizes the importance of maintaining clinical awareness of rare gastrointestinal metastatic sites during long-term surveillance of patients with HCC.

## 4. Conclusions

In conclusion, we report an extremely rare case of solitary colonic metastasis from hepatocellular carcinoma occurring 7 years after hepatic resection. The patient underwent successful surgical resection of the colonic metastatic lesion and remained alive without evidence of recurrence or additional metastasis at the 2-year follow-up. Although gastrointestinal metastasis from HCC is uncommon, clinicians should maintain vigilance for atypical metastatic sites during long-term surveillance, even in patients without intrahepatic recurrence.

Colonic lesions identified in patients with a history of HCC should be carefully evaluated, as they may represent metastatic disease rather than primary colorectal malignancy. A combination of imaging evaluation, endoscopic examination, pathological assessment, and immunohistochemical analysis is essential for accurate diagnosis. Surgical resection may be considered in carefully selected patients with isolated and resectable gastrointestinal metastases, particularly when intrahepatic disease is controlled and complete tumor removal is achievable.

Further studies, including systematic reviews of gastrointestinal metastases from HCC and molecular investigations into the mechanisms underlying unusual metastatic patterns, are needed to improve our understanding of disease progression and optimize the management of these rare cases.

## Author contributions

**Conceptualization:** Zhenxing Zhang, Kewei Ji.

**Data curation:** Guolin Zhang, Kelong Tao.

**Formal analysis:** Zhenxing Zhang, Guolin Zhang, Kelong Tao.

**Investigation:** Zhenxing Zhang, Shan Wang.

**Supervision:** Guangen Xu, Shan Wang.

**Writing – original draft:** Zhenxing Zhang, Shan Wang.

**Writing – review & editing:** Guangen Xu, Shan Wang.
